# Drug Repurposing and DNA Damage in Cancer Treatment: Facts and Misconceptions

**DOI:** 10.3390/cells9051210

**Published:** 2020-05-13

**Authors:** Eleni Sertedaki, Athanassios Kotsinas

**Affiliations:** Molecular Carcinogenesis Group, Lab Histology-Embryology, Faculty of Medicine, National Kapodistrian University of Athens, 11527 Athens, Greece; elenasertedaki@gmail.com

**Keywords:** drug repurposing, DNA damage, disulfiram, cancer

## Abstract

Drug repurposing appears to offer an attractive alternative in finding new anticancer agents. Their applicability seems to have multiple benefits, among which are the potential of immediate efficacy assessment in clinical trials and the existence of patient safety and tolerability evidence. Nevertheless, their effective application in terms of tumor-type targeting requires accurate knowledge of their exact mechanism of action. In this review, we present such a successful drug, namely Disulfiram (commercially known as Antabuse), and discuss its recently uncovered mode of anticancer action through DNA damage.

## 1. Introduction

Cancer or the “Pathology of the Century” is the most widely spread disease worldwide. It affects millions of people, constituting one of the main causes of mortality and morbidity in our growing old societies. In spite of the significant progress achieved in biomedical sciences in elucidating cancer biology and the evolvement of medical diagnostic and therapeutical methods, malignant diseases remain a major clinical problem. Established current treatment approaches such as surgery, chemotherapy, and radiotherapy have been proven effective in eradicating cancer cells, but they still have a devastating impact on normal human cells, while novel targeted therapies with minor side effects can be applied with success only on specific cases [1,2].

Creation of selective anticancer medicines, powerful against the different types of this heterogeneous disease, is the greatest challenge for the scientific community presently. In this effort, targeting cancer stem cells (CSCs) has recently emerged as an attractive option. However, the prohibitive costs and time needed not only for the identification of relevant target molecules (involved in CSCs survival) but also for the formulation and testing of new drugs as well as the failure of them in clinical practice place limits on research. At this point, drug repurposing turned out to be a novel promising alternative approach [1,2,3].

## 2. Drug Repurposing: The Example of Disulfiram (DSF)

Drug repurposing or drug repositioning is the new trend in cancer treatment. Its theory is based on the use of existing drugs on new targets. The application of such medications on malignant diseases is believed to have multiple benefits, among which are the potential of immediate efficacy assessment in clinical trials and the existence of patient safety and tolerability evidence [1,2].

In support of this effort, a huge attempt of screening large cohorts of patients on particular pharmacological therapies (preferably with a long follow-up period) has started, examining relative cancer morbidity and mortality rates in order to identify potential pleiotropic anticancer effects of drugs already in use for other therapeutic purposes. Towards this aim, an extensive recording of experiments, compounds, and cell lines that combined worldwide projects through sophisticated statistical analysis and further experimental verification have revealed feasible tumor biomarkers and potential anticancer drugs [1].

Nevertheless, acquisition of clinical and generally in vivo data is indicative but not sufficient to confirm a drug’s effects and efficiency. It is of paramount importance to clarify all of its mechanisms of action through an interdisciplinary approach before approving it especially as a cancer treatment modality [2].

The example of disulfiram (DSF) (commercially Antabuse) perfectly illustrates this need and highlights that superficial interpretation of experimental approaches can lead to erroneous conclusions and misconceptions. Particularly, DSF or tetraethylthiuram disulfide has been widely used for more than six decades for the treatment of alcohol dependence. Interestingly, the aforementioned patient screening demonstrated lower cancer mortality rates of Danish DSF users at the population level (data derived from a large cohort of Danish patients). In particular, according to epidemiological data, current and continuing DSF users had a cancer survival benefit compared to past users and the general population [1].

This observation implied that DSF may be toxic to cancer cells, and it was proposed as a potential anticancer treatment. This effect was also correlated with mutations of 16q chromosome, which encodes metal-binding proteins. Knocking out of relevant genes on glioma cell line SF295 (resistant to DSF) allowed for DSF’s copper-dependent activation that finally led to restriction of cell growth. As 16q chromosomal arm deletion is common in multiple cancer types, it could become a reliable biomarker to identify tumor sensitivity to DSF cytotoxicity [1].

In terms of mechanism, DSF’s cancer toxicity has been considered mediated by direct acetaldehyde dehydrogenase (ALDH) inhibition potentiated by copper ions supply. ALDH is an enzyme expressed by various cell types including cancer stem cells. Its main function is elimination of acetaldehydes and other toxic metabolic products, the accumulation of which can drive cells to death. In addition, it is recognized as a biomarker involved in cancer cell survival and metastatic spread. Given its crucial role, it could serve as an appealing therapeutic target to eliminate cancer cells. Furthermore, according to several studies, DSF seemed able to restrain CSC survival. Starting with a report on DSF toxicity on cancer breast cells, later ones expanded its toxic effects in different cancer types such as prostate and lung cancer [1,3].

Notably, Taconi and collaborators reported on the sensitivity of *BRCA1*- and *BRCA2*-deficient cells to DSF, the findings of which suggested DSF as a direct ALDH inhibitor and cancer therapeutic factor [3]. Given that *BRCA1* and *BRCA2* genes encode proteins that participate in homologous recombination-mediated DNA repair, mutations of these genes, met in various cancer types such as breast, ovarian, prostate, and pancreatic cancer, render malignant cells susceptible to agents inducing DNA defects, such as those provoked by acetaldehydes. Based on this, the researchers demonstrated that cell growth in a *BRCA1*- and/or *BRCA2*-deficient cellular environment was sensitized by acetaldehyde accumulation in human and mouse cell cultures. Extending on this, the authors also supported that the addition of DSF as an ALDH inhibitor led to a reduction in cell survival, an effect potentiated by copper anions supplementation, and that, consistently, *ALDH2* gene-deficient cells were sensitive to acetaldehydes. Yet, long-term incubation of cell cultures with DSF failed to eliminate cell survival after a certain point, attributing it to a lower cell membrane penetration of the compound [3].

However, despite this particular observation, the fact that the addition of DSF could not inhibit cell proliferation after a certain point and that the reversal of this condition was achieved by culture medium restoration raises questions about Taconi et al.’s conclusions, particularly on in vivo toxic mechanism of DSF [2]. This more careful view in combination with a groundbreaking experimental attempt led to the elucidation of DSF’s true mechanisms of action. Skrott et al. reproduced previous experiments trying to elucidate DSF actual mechanisms and uncovered its exact mechanism of action.

First of all, using the same H1299 cell line, they confirmed that *BRCA*-deficient models were more vulnerable to DSF treatment than proficient ones [2,4]. Furthermore, the investigators focused on tracing DSF metabolic byproducts. Interestingly, according to multiple reports, DSF is an extensively metabolized molecule, both in vivo and in vitro, ending up in the formation of a number of metabolites. Even though this knowledge is already widely accepted in other research fields, cancer-related studies neglected this data for years, focusing on direct ALDH inhibition by DSF. In particular, DSF is rapidly metabolized to diethyldithiocarbamate (DDTC), which is then converted to S-methyl-N,N-diethyldithiocarbamate (DETC) and S-methyl-N,Ndiethyldithiocarbamate (Me-DDTC). Furthermore, P450-catalyzed oxidation of DETC and Me-DDTC leads to the formation of DETC-sulfoxide (DETC-SO), S-methyl-N,N-diethylthiocarbamate-sulfoxide (Me-DTC-SO), and S- methyl-N,N-diethylthiocarbamate-sulfone (Me-DTC-SO2). These metabolites are actually directly involved in ALDH inhibition. Consistent with these findings, P450 inhibition leaves ALDH uninhibited, proving that downstream DSF metabolites and not DSF itself are the ultimate in vivo ALDH inhibitors [4].

With this knowledge as a starting point, Skrott et al. managed to identify the ultimate DSF anticancer metabolite: the copper containing molecule ET (CuET), which was able to inhibit cancer cell proliferation [2].

CuET is a DTC copper complex that forms spontaneously in vivo and in cell cultures. It is found in higher concentrations in tumor counterparts compared to corresponding normal liver and brain tissues, while formation of CuET is enhanced in humans undergoing DSF treatment for alcoholism. Particularly, Skrott et al. detected CuET in DSF-treated cell cultures without addition of copper and further confirmed its formation both in vivo and in vitro. They proved that supplementation of copper anions empowered its formation, exerting a further deteriorating effect on cancer cells, exactly at the point when addition of DSF could not affect cell survival. This is the phenomenon mistakenly—so far—attributed to limited cell membrane penetration by DSF, while it actually reflects copper anions’ concentration in culture media. Consistently, chelation of copper by addition of bathocuproinedisulfonic acid (BCDS), a metal chelator, can diminish CuET levels [2,4].

Regarding its mechanism of action, CuET causes aggregation of Nuclear protein localization protein 4 (NPL4), primarily with the help of copper anions. NPL4 is an essential cofactor of p97/VCP segregase, a complex responsible for the repair of DNA double strand breaks. Its P97 component, which extracts ubiquinated substrates, is immobilized via NPL4 connection, excluding chromatin, rather than accumulating in specific nuclear areas. Consistent with previous findings, sedation of cooper ions by BCDS suppresses the CuET effect on NPL4, explaining the already observed reversal of DSF effect by BCDS [2,4].

Additional research by Skrott et al. revealed that CuET treatment induces accumulation of single-stranded DNA (ssDNA) decelerating replication fork progression. Subsequent DNA damage, occurring mostly in the S phase, activates homologous recombination DNA repair pathway normally, so that ssDNA stretches are covered with replication protein A (RPA), which activates the ATRIP-ATR-CHK1 signaling pathway involving also BRCA1 and BRCA2. Thus, obstruction or genetic deficiency of Ataxia telangiectasia and Rad3-related kinase (ATR kinase) reverses such replication stress cell responses, inducing genome instability and cell death. Therefore, Skrott et al. demonstrated that CuET traps ATR kinase within NPL4 aggregates, further enhancing replication stress [5].

Moreover, the investigators observed that NPL4-CuET-induced aggregates trigger cellular heat shock responses through activation of proteotoxic stress-related proteins, including HSP70, SUMO2/3, polyubiquitin chains, and TDP-43, offering a meaning explanation for the previously observed proteasome-related DSF toxicity [2,5].

In addition, Skrott et al. examined the hypothesis of ALDH inhibition by DSF. Using two human cancer cell lines, K562 and A549, they measured total ALDH activity, revealing that ALDH inhibitors were neither DSF nor CuET but Me DTC SO and DEAB, two metabolites without toxic impact on cells already known in the literature. These molecules are evidently involved in ALDH inhibition, but they are not able to obstruct cell growth themselves. Suppression of cell proliferation along with a reduction of ALDH activity in the long term occurs due to an overall accumulation of toxic metabolites rather than the impact of any particular substance, as Skrott et al. demonstrated. In support of this, if growth restriction was mediated enzymatically, it would take place within a few hours, not after a long exposure to the harmful factor. As expected, decreased cell fitness is correlated with increased cell permeability and increased cell death [2].

Overall, the case of “disulfiram misconception” constitutes an example pointing that thorough research towards a deep understanding of molecular mechanisms is crucial when selecting targeted therapies, especially before choosing drugs to reposition [4]. DSF has been used for years with success against alcohol abuse. When suggested as a potential cancer treatment, the classical notion of direct ALDH inhibition by DSF itself prevailed without further interrogation, even underestimating existing knowledge in other fields. Initial preclinical studies were conducted in cell cultures and in vivo models without detailed experimental verification of findings at molecular level. To make matters worse, ambiguous results were explained in accordance with established beliefs while robust findings in other scientific fields remained overlooked for long. Thus, DSF was considered to eradicate cancer stem cells through direct inhibition of the ALDH pathway becoming an attractive factor against numerous cancer types expressing this biomarker. However, further investigation led to the discovery of unexplored DSF metabolites and to the revelation of different mechanisms of action that we actually ignored until recently [2,4,5].

It is of paramount importance though to underline that new data have yet elucidated partially DSF mechanisms of action. The discovery of new cellular targets and signaling pathways bore new questions that remain to be explored. Especially the involvement of DSF in cellular replication stress responses is quite sophisticated and requires further investigation in order to be deeply understood. Skrott et al. address a number of such issues for future research in their last report, underlining that there is still much to learn [5].

## 3. Conclusions

We have to recognize that existing drugs establish starting points for the development of new therapies when targets for old drugs are discovered. However, this selection should be undertaken with care and cautiousness, taking into account patient sensitivity, intolerance, and quality of life. Appropriately targeted therapies need to offer confirmed benefits, minimizing adverse effects. In this context, epidemiological and clinical data should be critically assessed. They are very valuable in indicating the potential of various medicines driving scientific research towards specific biological targets, but efficient application of a medication requires the elucidation of its mechanism of action and a detailed tracing of its molecular targets. In this context, an interdisciplinary approach is of vital significance. Collaboration of different scientific fields will contribute to appropriate interpretation of clinical observations, to deciphering molecular mechanisms involved in cancer cells’ survival, and to a thorough understanding of drugs’ effects at cellular level. The long way against cancer treatment must be based on robust evidence, not on misconceptions.
